# Non-invasive assessment of cardiac function and pulmonary vascular resistance in an canine model of acute thromboembolic pulmonary hypertension using 4D flow cardiovascular magnetic resonance

**DOI:** 10.1186/1532-429X-16-23

**Published:** 2014-03-13

**Authors:** Alejandro Roldán-Alzate, Alex Frydrychowicz, Kevin M Johnson, Heidi Kellihan, Naomi C Chesler, Oliver Wieben, Christopher J François

**Affiliations:** 1 Department of Radiology, Clinical Science Center, University of Wisconsin - Madison, 600 Highland Avenue, Madison, Wisconsin 53792-3252, USA; 2 Department of Medical Physics, University of Wisconsin – Madison, Madison, WI, USA; 3 Klinik für Radiologie und Nuklearmedizin - Campus Lübeck, Lübeck, Germany; 4 School of Veterinary Medicine, University of Wisconsin – Madison, Madison, WI, USA; 5 Department of Biomedical Engineering, University of Wisconsin – Madison, Madison, WI, USA

**Keywords:** Pulmonary hypertension, Pulmonary vascular resistance, Heart function, 4D flow cardiovascular magnetic resonance, Thromboembolic pulmonary hypertension, Canine model

## Abstract

**Background:**

The purpose of this study was to quantify right (RV) and left (LV) ventricular function, pulmonary artery flow (Q_P_), tricuspid valve regurgitation velocity (TRV), and aorta flow (Q_S_) from a single 4D flow cardiovascular magnetic resonance (CMR) (time-resolved three-directionally motion encoded CMR) sequence in a canine model of acute thromboembolic pulmonary hypertension (PH).

**Methods:**

Acute PH was induced in six female beagles by microbead injection into the right atrium. Pulmonary arterial (PAP) and pulmonary capillary wedge (PCWP) pressures and cardiac output (CO) were measured by right heart catheterization (RHC) at baseline and following induction of acute PH. Pulmonary vascular resistance (PVR_RHC_) was calculated from RHC values of PAP, PCWP and CO (PVR_RHC_ = (PAP-PCWP)/CO). Cardiac magnetic resonance (CMR) was performed on a 3 T scanner at baseline and following induction of acute PH. RV and LV end-diastolic (EDV) and end-systolic (ESV) volumes were determined from both CINE balanced steady-state free precession (bSSFP) and 4D flow CMR magnitude images. Q_P_, TRV, and Q_S_ were determined from manually placed cutplanes in the 4D flow CMR flow-sensitive images in the main (MPA), right (RPA), and left (LPA) pulmonary arteries, the tricuspid valve (TRV), and aorta respectively. MPA, RPA, and LPA flow was also measured using two-dimensional flow-sensitive (2D flow) CMR.

**Results:**

Biases between 4D flow CMR and bSSFP were 0.8 mL and 1.6 mL for RV EDV and RV ESV, respectively, and 0.8 mL and 4 mL for LV EDV and LV ESV, respectively. Flow in the MPA, RPA, and LPA did not change after induction of acute PAH (p = 0.42-0.81). MPA, RPA, and LPA flow determined with 4D flow CMR was significantly lower than with 2D flow (p < 0.05). The correlation between Q_P_/TRV and PVR_RHC_ was 0.95. The average Q_P_/Q_S_ was 0.96 ± 0.11.

**Conclusions:**

Using both magnitude and flow-sensitive data from a single 4D flow CMR acquisition permits simultaneous quantification of cardiac function and cardiopulmonary hemodynamic parameters important in the assessment of PH.

## Background

Pulmonary hypertension (PH) is an ultimately fatal disease characterized by an abnormal increase in the mean pulmonary artery pressure (mPAP) that ultimately leads to right ventricular (RV) failure [1]. The initial evaluation of patients with suspected PH includes non-invasive imaging with echocardiography because of its ability to non-invasively estimate pulmonary artery pressures, pulmonary vascular resistance (PVR) and cardiac function [2]. Depending upon the results of the initial assessment with echocardiography, further imaging work-up to identify the underlying cause and determine appropriate management of PH may include ventilation/perfusion scintigraphy in patients with known or suspected chronic thromboembolic PH, computed tomography to assess for diffuse lung disease, and invasive right heart catheterization (RHC) [3] to confirm the diagnosis. Currently, RHC is considered the reference standard for assessing PH patients and providing definite diagnosis, because the categorization of the type of PH is based on the observed pulmonary artery pressures (mPAP > 25 mmHg), pulmonary capillary wedge pressure (PCWP < than 15 mmHg) and pulmonary vascular resistance (PVR > 3WU) [4].

Cardiovascular magnetic resonance (CMR) is increasingly being used to monitor pulmonary hemodynamics and cardiac function in patients with PH. Flow-sensitive CMR sequences can be used to measure pulmonary artery flow (Q_P_) flow and TRV, and thereby estimate PVR (PVR_RHC_ ∝ TRV/Q_p_) using a method analogous to that used by Abbas *et al.* for echocardiography [5]. Using this model, TRV is used as a surrogate of peak systolic pulmonary arterial pressure and reflects the trans-tricuspid gradient. With respect to cardiac size and function, CMR is considered the gold standard for quantification of left [6-8] and right [9-11] ventricular size and function with time-resolved “CINE” balanced steady-state free precession (bSSFP) imaging due to its high reproducibility.

In this study we have investigated an alternative CMR approach to assess flow and ventricular function using a single 4D flow MR sequence that uses a radially undersampled, time-resolved, 3-dimensional, 3-directionally velocity-encoded imaging scheme [12]. Ventricular size and function were measured using the time-resolved magnitude images and compared with values obtained using standard CINE bSSFP and two-dimensional flow-sensitive (2D flow) imaging. Tricuspid valve, pulmonary artery and aorta flow was quantified using the time-resolved phase images and compared with ventricular stroke volumes obtained using volumetric methods. In addition, the ratio of TRV/Q_P_ was correlated to PVR_RHC_ to determine if this method could also be used to estimate PVR non-invasively. A benefit of using the same sequence for measuring cardiac chamber volumes and flow includes an overall shortened examination acquisition time (particularly in cases where numerous flow measurements are required). In addition, a free-breathing acquisition is beneficial in patients who have difficulty with the numerous breath holds required using standard cardiac MR sequences. Although not explored in this study, 4D flow MR has been shown to be of use in assessing additional hemodynamic characteristics of normal and abnormal flow in a variety of cardiovascular diseases [13].

## Methods

### Acute canine thromboembolic pulmonary hypertension model

All studies were approved by the institutional local animal care and use committee (RARC). Acute PAH was induced by injection of micro-beads (150–500 μm) in the right atrium and ventricle in six adult female beagles (8.3 ± 2.4 kg). Dogs were anesthetized with propofol (10 mg/kg body weight), intubated, and maintained under anesthesia with isoflurane (1 to 3%) with 100% oxygen. During anesthesia, ventilation was adjusted to keep end-tidal CO_2_ within normal limits (30–50 mmHg). A femoral arterial catheter was inserted and systemic arterial pressure (SAP) and arterial blood gases were monitored. The femoral and external jugular veins were catheterized for RHC access, delivery of emboli, delivery of contrast for angiography and blood sampling.

While maintaining the dog under anesthesia, CMR and RHC were repeated twice, the first prior to induction of PH to obtain baseline data, and the second repeating all acquisitions after successful induction of thromboembolic PH (confirmed by > twofold increase in baseline mPAP). The dogs were euthanized according to the RARC protocol after the post-embolization CMR study.

### Right heart catheterization

After induction of anesthesia, dogs were granted a resting period of approximately 30 minutes to ensure adaptation of the circulation to anesthesia. Baseline pulmonary arterial pressure (PAP), pulmonary capillary wedge pressure (PCWP) and right ventricular cardiac output (Q_P_) were measured. Measurements were made using a 7.5-French, fluid-filled catheter connected to an analog pressure recorder. PVR [in Woods units (WU)] was calculated using the formula, analogous to Ohm’s law for electrical circuits: PVR_RHC_ = ∆P/Q_P_, where ∆P is the trans-pulmonary pressure gradient (∆P = mPAP - PCWP) [14] and Q_P_ is the flow in the pulmonary artery measured by thermodilution.

### Magnetic resonance imaging

CMR measurements were performed prior to and following induction of PH resulting in a total of 12 measurements on a 3.0 T clinical systems (MR750, GE Healthcare, Waukesha, WI). Contiguous axial CINE bSSFP slices were obtained covering the entire heart. Parameters for CINE bSSFP imaging included: 310 × 190 mm field of view, 224 × 192 acquisition matrix, 7 mm slice thickness, 0 mm gap, ±125 kHz bandwidth, 45° flip angle, TR/TE = 3.1/1.1 ms (fractional echo readout), and a prospectively gated, k-space segmented acquisition (12 views per segment), for an acquired temporal resolution of 37 ms. 20 temporally interpolated time frames were reconstructed at each slice location. Between 13 and 16 slices were acquired depending on subject anatomy. Each slice was acquired within an 8 to 10 second time interval of suspended ventilation.

2D and 4D flow CMR were performed following the administration of 0.1 mmol/kg of Gd-based intravenous contrast (gadobenate dimeglumine, Bracco Diagnostics, Inc., Princeton, NJ). A standard 2D flow CMR sequence was used to assess flow through the main, right, and left pulmonary arteries (MPA, RPA and LPA, respectively). Parameters for 2D flow imaging were: 160-220 × 160-176 mm field of view, 256 × 128 acquisition matrix, 5 mm slice thickness, TR/TE = 5.53-5.93/3.17-3.44 ms, and a prospectively gated, k-space segmented acquisition. Temporal resolution was 22.11-35.44 ms and data were interpolated to 20 reconstructed time frames for each location. 2D flow CMR acquisitions were performed during a breath-hold at end-expiration.

4D flow CMR was performed using a previously described 3D radial sequence [15]. Parameters were: imaging volume = 320 × 320 × 220 mm, readout length = 256 samples, TR/TE = 6.7/2.4 ms, flip angle 10-20°, spatial resolution = 1.3 mm isotropic. Retrospective ECG-gating was used. Respiratory triggering with bellows allowed for breathing compensation with an acceptance rate of 50%, resulting in a scan duration of 10–12 min during free breathing. Data were retrospectively sorted into 20 time frames according to their position in the cardiac cycle. Subsequently, image reconstruction was performed utilizing a compressed sensing reconstruction and a temporal filter for view sharing [16]. To minimize the number of slices needing manual segmentation for measuring ventricular volumes, three contiguous slices were averaged. In addition, time-resolved 4D flow CMR magnitude images were reformatted into the left-ventricular short-axis (SA) orientation using home-built MatLab software, for a second, more convenient LV segmentation.

### CMR analysis

#### RV and LV function

**Figure 1:**
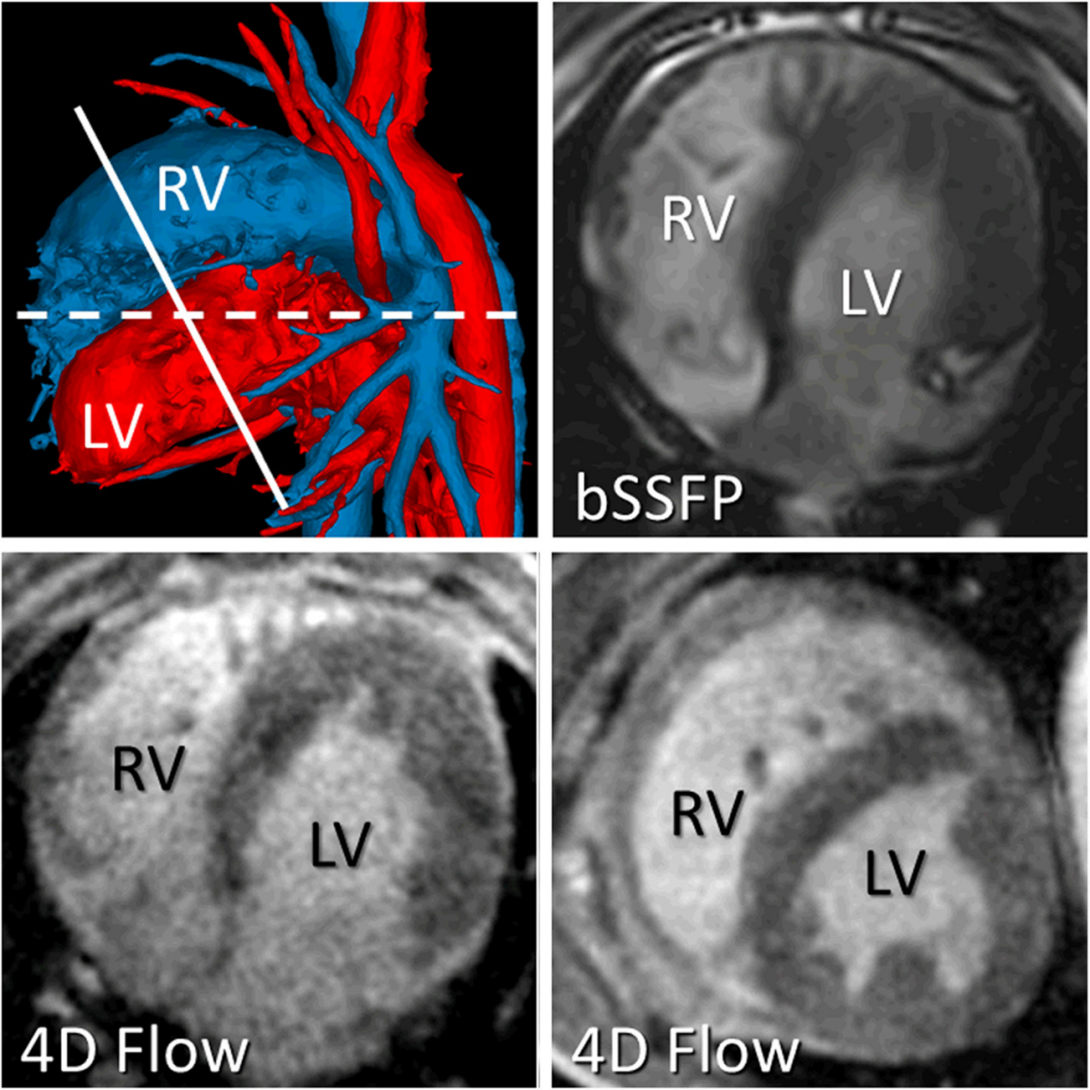
**Surface-shaded angiographic image (top left) from 4D flow MRI dataset revealing right- (blue) and left- (red) sided circulations.** Right (RV) and left (LV) ventricular volumes were calculated from bSSFP (top right) and 4D flow MRI magnitude (bottom left) images in the axial orientation (dashed line). In addition, the time-resolved 4D flow MRI magnitude dataset was reformatted into the LV short-axis orientation (bottom right and indicated by solid line in top left).

RV and LV end-diastolic (EDV) and end-systolic (ESV) volumes were determined from manually segmented contours of end-diastolic and end-systolic bSSFP and 4D flow CMR images (Figure 1), respectively. Segmentation of bSSFP images was accomplished using ReportCard (GE Healthcare, Waukesha, WI). Segmentation of 4D flow CMR images was conducted using Osirix (Pixmeo, Geneva, Switzerland). Stroke volume (SV = EDV-ESV) and ejection fraction (EF = SV/EDV) were also determined for both bSSFP and 4D flow CMR.

#### Flow quantification

Analysis of the 2D flow CMR data was performed with CV flow (Medis, Leiden, the Netherlands). The MPA, RPA, and LPA were manually segmented at each phase of the cardiac cycle. Net flow per heart beat was recorded for each location. Average velocity was recorded for the MPA only. PVR was estimated from 2D flow and 2D CINE bSSFP data using the following equation PVR_CMR_ (WU) = 19.38 – [4.62 · lnPA average velocity] – [0.08 · RVEF] [17].

**Figure 2:**
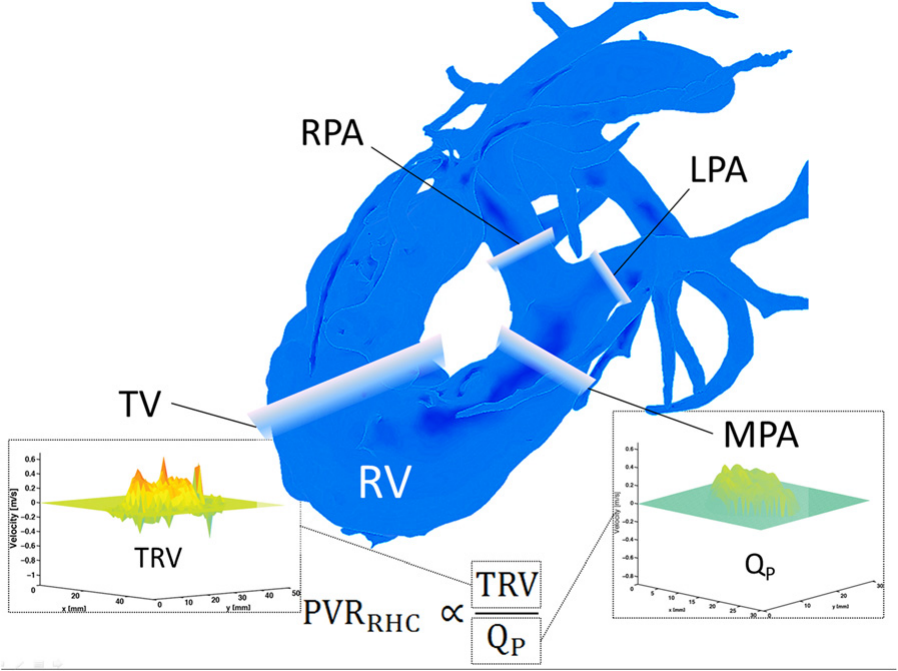
**Surface-shaded angiographic image from 4D flow MRI dataset indicating locations of flow quantification in the right-sided circulation.** Flow analysis was performed at the level of the tricuspid valve (TV), main pulmonary artery (MPA), right pulmonary artery (RPA), and left pulmonary artery (LPA). The ratio of the peak tricpuspid regurgitation velocity (TRV) to flow through the MPA (Q_P_) was correlated to pulmonary vascular resistance at right heart catheterization (PVR_RHC_).

**Figure 3:**
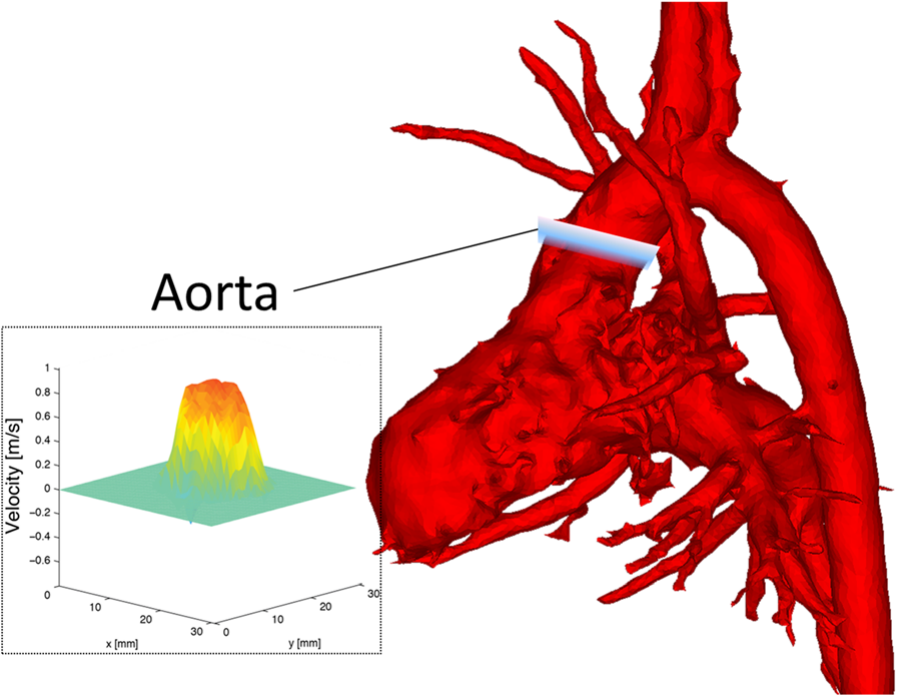
Surface-shaded angiographic image from 4D flow MRI dataset indicating location of flow analysis in the ascending aorta.

Quantitative flow analysis of the 4D flow CMR datasets was performed with a previously described Matlab-based home built software (The MathWorks, Natick, MA, USA) [18]. Two-dimensional cutplanes were manually placed perpendicular to the direction of the flow in the tricuspid valve, MPA, RPA, LPA (Figure 2) and aorta (Figure 3) using Ensight (CEI, Apex, NC, USA). Specifically, the tricuspid valve plane was generated using flow streamlines, which allowed for localization of the peak tricuspid regurgitation velocity with no need for annular motion compensation. From these analyses, we recorded (a) the peak tricuspid valve regurgitation velocity (TRV) for the generated two-dimensional tricuspid valve cutplane; (b) net flow, time-to-peak flow, and time-to-peak acceleration in the MPA, RPA, and LPA; and (c) net flow in the aorta. To assess the internal consistency of the flow measurements, we determined the relative flow through the RPA and LPA, as a percentage of MPA flow, and the ratio of pulmonary (Q_P_) to aorta (Q_S_) flow.

### Statistical analysis

Values are reported as mean ± standard deviation. Differences between flow (2D and 4D) and CINE bSSFP and between the two flow techniques were assessed using Bland-Altman analysis. Linear regression analysis was used to determine the Pearson correlation coefficients between TRV/Q_P_ and PVR_RHC_ and mPAP. A linear regression equation was derived to calculate PVR_4Dflow_ from the imaging parameters. Bland-Altman analysis was used to assess the differences between the PVR_RHC_ and PVR_4Dflow_ and PVR_CMR_. Values obtained prior to and following induction of PH were compared used a paired Student’s t-test. P-values less than 0.05 were considered statistically significant.

## Results

### RV and LV function

Mean values for RV and LV volumes using CINE bSSFP and 4D flow CMR are summarized in Table 1. Differences in RV EDV, ESV, and SV were not significant (P > 0.05). LV EDV determined with both axial and short-axis reconstructions did not significantly differ from that measured using axial CINE bSSFP (P > 0.05). LV ESV and SV were significantly larger with 4D flow CMR than CINE bSSFP using both axial and short-axis reconstructions (P < 0.05). LV EDV and SV were smaller (P = 0.02 and 0.001, respectively) and LV ESV was larger (P = 0.10) when calculated from the short-axis reconstructions than the axial reconstructions.

**Table 1:** Summary of right (RV) and left (LV) ventricular volumes measured from standard CINE balanced steady-state free precession (bSSFP) and 4D flow CMR magnitude images (PC-VIPR)

	**CINE bSSFP**	**4D flow CMR**	
**RV**	**Axial**	**Axial**	
EDV (mL)	34.5 ± 8.1	35.3 ± 10.2	
ESV (mL)	19.6 ± 5.8	21.2 ± 6.8	
SV (mL)	14.8 ± 3.1	14.1 ± 4.1	
EF	0.44 ± 0.07	0.40 ± 0.06	
CO (L/min)	1189 ± 286	1145 ± 417	
**LV**	**Axial**	**Axial**	**Short-axis**
EDV (mL)	22.3 ± 5.4	23.8 ± 6.5	23.0 ± 6.2
ESV (mL)	9.1 ± 3.6	12.5 ± 4.2^1^	13.1 ± 3.8^1^
SV (mL)	13.1 ± 3.6	11.3 ± 3.0^1^	9.9 ± 3.4^1,2^
EF	0.59 ± 0.11	0.48 ± 0.07^1^	0.43 ± 0.09^1,2^
CO (L/min)	1059 ± 358	907 ± 295^1^	798 ± 322^1,2^

LV volumes were determined from both axial and short-axis (SA) images. Data from pre- and post-embolization CMR studies are combined.

^1^P < 0.05 with respect to CINE bSSFP. ^2^P < 0.05 with respect to axial 4D flow CMR. *Abbreviations* – *EDV* End-diastolic volume, *ESV* End-systolic volume, *SV* Stroke volume, *EF* Ejection fraction, *CO* Cardiac output.

### Flow quantification

#### Pulmonary arteries

Results of the flow analysis in the MPA, RPA and LPA are summarized in Tables 2 and 3. One pre- and one post-embolization 4D flow datasets were excluded due to inadequate imaging quality. Flows were significantly higher with 2D flow than with 4D flow. Relative flow through the RPA and LPA was 61% and 39%, respectively, with 4D flow and 59% and 41%, respectively, with 2D flow. The average flow through the RPA and LPA combined (12.6 ± 3.5 mL/cycle with 4D flow and 18.4 ± 6.4 mL/cycle with 2D flow) was not significantly different from the flow measured through the MPA (12.0 ± 3.9 mL/cycle with 4D flow, P = 0.96, and 18.9 ± 3.1 mL/cycle with 2D flow, P = 0.70).

**Table 2:** Summary of pulmonary artery flow quantification pre- and post-embolization using 2D and 4D flow techniques

	**2D Flow**				**4D Flow**			
	**MPA**	**RPA**	**LPA**	**RPA + LPA**	**MPA**	**RPA**	**LPA**	**RPA + LPA**
Pre + Post	18.9 ± 4.5^1,2^	10.7 ± 3.2^2^	7.7 ± 3.8^2^	18.4 ± 6.4^2^	12.0 ± 3.9^1^	7.6 ± 2.2	4.8 ± 2.1	12.6 ± 3.5^1^
Pre	19.25 ± 4.5^1,2^	11.1 ± 2.9^2^	8.0 ± 2.9	19.1 ± 5.4^2^	12.2 ± 3.3^1^	7.4 ± 2.3	4.8 ± 2.7	12.5 ± 4.0
Post	18.6 ± 5.0^1,2^	10.4 ± 3.8^2^	7.3 ± 4.8	17.7 ± 7.7^2^	11.8 ± 4.6	7.8 ± 2.3	4.9 ± 1.3	12.7 ± 3.5

All values are mean flow per heart beat ± standard deviation (mL). ^1^ – P < 0.05 for MPA flow compared to Cine 2D bSSFP right ventricular stroke volume. ^2^ – P < 0.05 compared to 4D flow. Differences in flow pre- and post-embolization were not significant (P ≥ 0.05). *Abbreviations* – *MPA* Main pulmonary artery, *RPA* Right pulmonary artery, *LPA* Left pulmonary artery.

**Table 3:** Summary of time-to-peak flow and acceleration in main, right and left pulmonary arteries (MPA, RPA, and LPA, respectively) measured from 4D flow data

	**TTP flow (ms)**			**TTP acceleration (ms)**		
	**MPA**	**RPA**	**LPA**	**MPA**	**RPA**	**LPA**
Pre + Post	279 ± 170	298 ± 149	305 ± 145	219 ± 161	238 ± 150	223 ± 141
Pre	339 ± 176	337 ± 161	351 ± 136	261 ± 162	270 ± 159	234 ± 149
Post	230 ± 163	259 ± 144	248 ± 149	184 ± 167	206 ± 152	210 ± 147

Differences in TTP flow and acceleration pre- and post-embolization were not significant (P ≥ 0.05).

Comparing flow data pre- and post-embolization, the differences in MPA, RPA, and LPA flows were not significant. Although there was a trend toward faster time-to-peak flow and time-to-peak acceleration values post-embolization in the pulmonary arteries, these changes were not significant (Table 3). Time-to-peak acceleration was shorter post-embolization than pre-embolization, although this difference was not significant (Table 3).

#### Aorta

The average aortic flow (Q_S_) using 4D flow CMR was 11.6 ± 4.0 mL/cycle (P = 0.06 with bSSFP and 0.08 with 4D flow CMR volumetry). The mean difference between Q_S_ with 4D flow CMR and LV SV calculated from 4D flow CMR was -0.7 mL. The difference between Q_S_ and MPA flow (Q_P_) calculated from 4D flow CMR phase-sensitive data was not statistically significant (P = 0.30). Furthermore, the average Q_P_/Q_S_ was 0.96 ± 0.11 (range: 0.87-1.21). Differences in aortic flow pre- and post-embolization, 12.0 ± 3.8 mL/cycle and 11.1 ± 4.4 mL/cycle, respectively, did not reach statistical significance (P = 0.81).

### PVR

**Figure 4:**
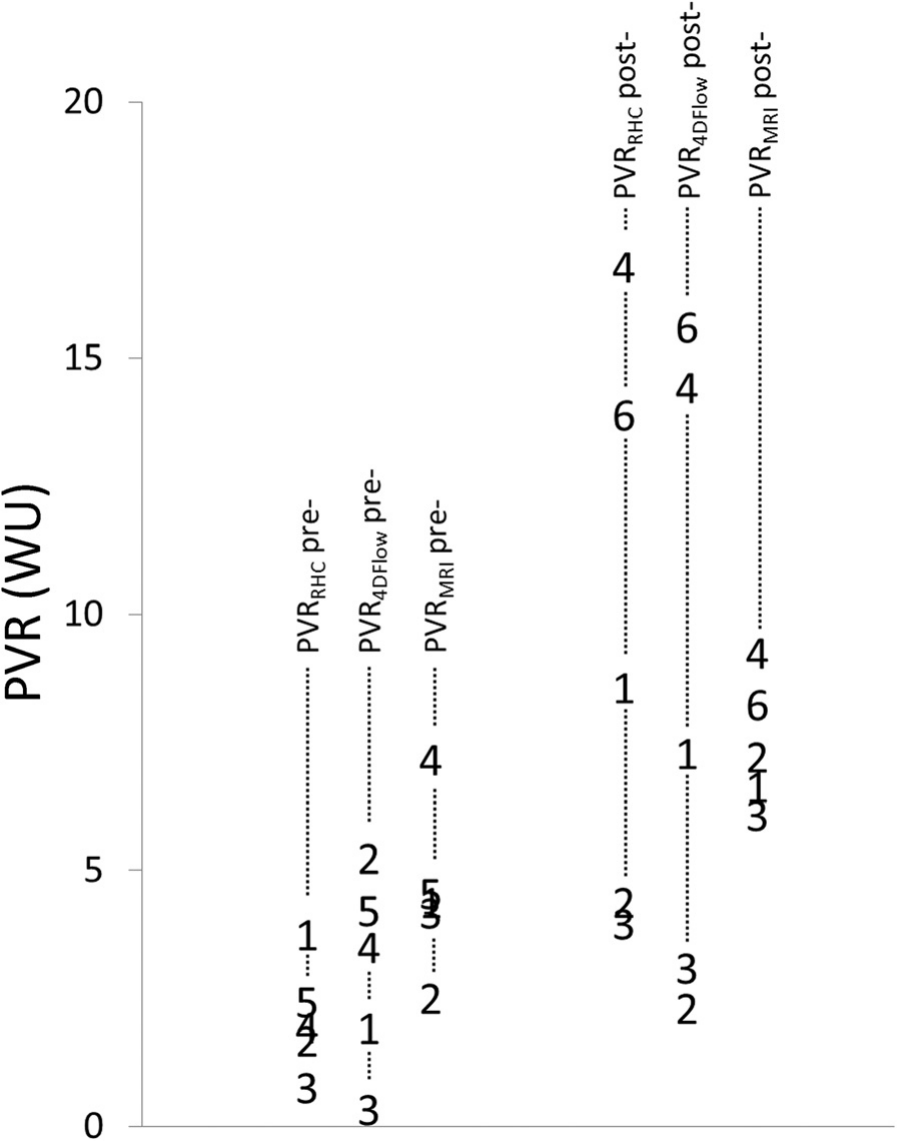
**Scatter-plot of PVR**_**RHC**_**, PVR**_**4Dflow **_**and PVR**_**MRI **_**pre- and post-embolization.** PVR_RHC_ and PVR_MRI_ increased post-embolization in all cases. In one case, PVR_4Dflow_ was lower post-embolization than pre-embolization, presumably due to lack of an increase in tricuspid regurgitation (TR) jet velocity or incorrect selection of the peak TR velocity.

**Figure 5:**
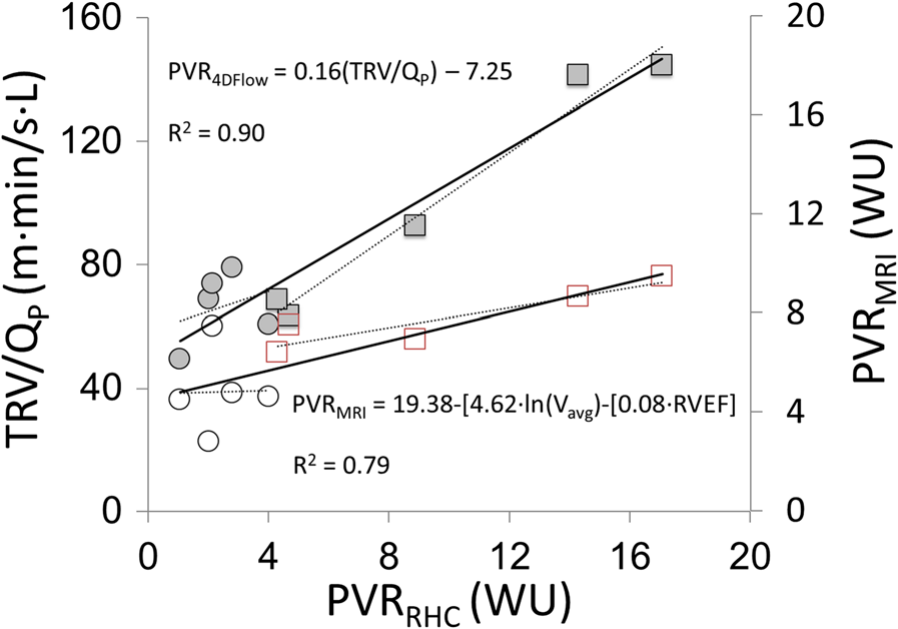
**Derivation of PVR**_**MRI **_**equation from the linear regression between TRV/ Q**_**P **_**and PVR**_**RHC **_**(PVR**_**MRI**_ **= 0.16(TRV/ Q**_**P**_**) – 7.25).**

**Figure 6:**
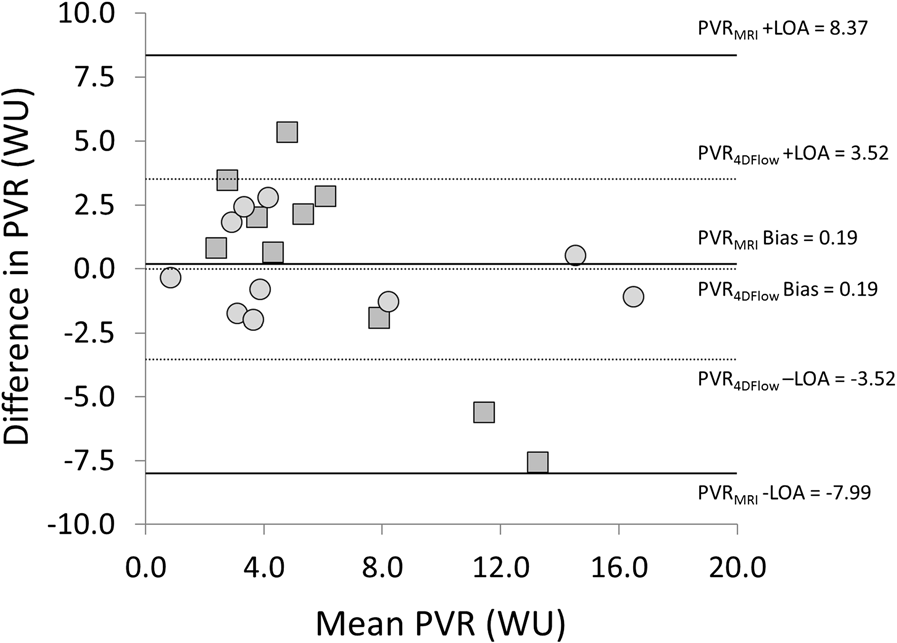
Bland-Altman plot for comparison of PVR calculated from RHC measurements and estimated from 4D Flow MRI data.

Tricuspid regurgitation was detected in all canines pre- and post-embolization, enabling calculation of PVR_CMR_ in all cases. PVR_RHC_ values pre- and post-embolization were 2.4 ± 0.9 WU and 9.8 ± 5.7 WU, respectively (Figure 4). The Pearson correlation coefficient between TRV/Q_P_ and PVR_RHC_ was 0.95 for combined pre- and post-embolization data (Figure 5). When analysing the data pre- and post-embolization separately, the correlation between TRV/Q_P_ and PVR_RHC_ was higher post-embolization (R = 0.99) than pre-embolization (R = 0.26). The Pearson correlation coefficient between TRV/Q_P_ and mPAP was 0.65. The equation for calculating PVR_4Dflow_ using the combined data was PVR_4Dflow_ (WU) = 0.16(TRV/Q_P_) – 7.25. Using this equation, PVR_4Dflow_ values pre- and post-embolization were 3.3 ± 1.8 WU (P = 0.35 for PVR_4Dflow_ vs. PVR_RHC_) and 9.0 ± 6.2 WU (p = 0.15 for PVR_4Dflow_ vs. PVR_RHC_), respectively (Figure 4). In one case, PVR_4Dflow_ was lower post embolization. The mean difference between PVR_4Dflow_ and PVR_RHC_ was 0 (WU) with positive and negative levels of agreement of 3.52 and -3.52 (WU), respectively (Figure 6).

Using 2D flow and 2D CINE bSSFP, PVR_CMR_ was 5.0 ± 1.6 (WU) pre-embolization (P = 0.051 with PVR_RHC_ and 0.21 with PVR_4Dflow_) and 7.5 ± 1.3 (WU) post-embolization (P = 0.38 with PVR_RHC_ and 0.66 with PVR_4Dflow_) (Figure 4). PVR_CMR_ increased post-embolization in all cases. The Pearson correlation coefficients between PVR_CMR_ and PVR_RHC_ was 0.78 for combined pre- and post-embolization data, 0.03 for pre-embolization data and 0.91 for post-embolization data.. The mean difference between PVR_CMR_ and PVR_RHC_ was 0.18 (WU) with positive and negative levels of agreement of 8.4 and -8.0 (WU), respectively.

## Discussion

In this study we demonstrated the feasibility of measuring RV and LV function and pulmonary vascular resistance from a single, free breathing 4D flow CMR sequence. RV and LV volumes were measured using the time-resolved magnitude data from the same 4D flow CMR acquisition. Using the three-directional velocity information, we found a strong correlation between the ratio of the peak tricuspid regurgitation velocity to the flow through the pulmonary arteries (TRV/Q_P_) and PVR determined at RHC, using an approach that is analogous to that used for echocardiography [5].

### RV and LV function

Cardiac magnetic resonance using breath-hold CINE balanced steady-state free precession imaging (bSSFP) is considered the gold standard for quantification of ventricular size and function [6-11]. However, in patients with PH, dyspnea can be severe, and the multiple breath-holds required to scan the entire heart can be difficult, or impossible, for many patients. An alternative approach is to use free-breathing acquisition methods with respiratory navigation or triggering to coordinate data acquisition with the respiration, as was done here with the 4D flow MR approach. Using a volumetric (3D) approach to assessing cardiac function would also be beneficial in that it would enable quantification of RV and left ventricular (LV) volumes simultaneously, which is important because the most common cause of pulmonary hypertension is left heart disease [19]. Quantification of LV and RV function using time-resolved 3D acquisitions has previously been demonstrated using both free-breathing and breath-hold bSSFP techniques [20,21]. Although free-breathing approaches to cardiac function analysis are longer than single breath-hold acquisitions, in our clinical experience, patients with PH frequently have difficulty holding their breath for even more than 10 seconds. Future studies in PH patients comparing these two approaches to whole-heart functional analysis are warranted. LV ESV was larger, and therefore LV SV was smaller, with time-resolved magnitude reconstructions from the 4D flow CMR dataset than with the standard 2D bSSFP acquisition. This may be partially related to the higher spatial resolution with the 4D flow CMR data, resulting in improved delineation of the blood-myocardial interface, which is frequently a challenge when using standard 2D bSSFP. The results from our study are encouraging in that the bias we observed using a 3D radially undersampled approach was of the same magnitude as previously published studies.

### Estimation of PVR

Currently, Doppler echocardiography is used clinically in patients with PH to estimate PVR from the ratio of the TRV to the velocity time integral in the right ventricular outflow tract (VTI_RVOT_), which is a surrogate of the flow through the pulmonary artery (Q_P_) [5]. Abbas *et al.* reported a strong linear correlation between TRV/VTI_RVOT_ and PVR_RHC_ and proposed calculating PVR from the following equation: PVR = 10 × TRV/VTI_RVOT_ in Woods units (WU). However, a potential important limitation of this approach is that Doppler ultrasound does not measure flow directly, but rather the velocity time integral which does not take into account the area of the vessel of interest. Furthermore, echocardiography-based methods of assessing PVR can be limited by body habitus or other anatomical factors obstructing the acoustic window. In contrast, CMR-based flow sequences can be used to measure flow, in addition to velocity, without the limitations of echocardiography. As a result, other investigators have used CMR to estimate PVR, generating similar models based on cardiac function and pulmonary artery flow. Garcia-Alvarez *et al.*[17] derived an equation for calculating PVR using the average MPA velocity and the RVEF. This model was derived in a cohort of human PH subjects [17] and subsequently validated in a second cohort and in a more recent canine model of acute and chronic thromboembolic PH [22]. Swift *et al.*[23] created a model to estimate PVR based on left atrial size (as an estimate of PCWP) and a linear regression equation for calculating mPAP from the interventricular septal angle and ventricular mass index. As with our study, limits of agreement for determining PVR using these models were also fairly wide, -6.0 to 4.9 WU (Garcia-Alvarez [17]) and -5.1 to 4.6 WU (Swift [23]). Although the bias we observed for PVR_CMR_ method using 2D flow and 2D CINE bSSFP was minimal (0.18 WU), the limits of agreement were fairly wide, wider than what we observed using the TRV/VTI_RVOT_ method and wider than what was reported by Garcia-Alvarez et al.

### Flow quantification with 4D flow

4D flow CMR has been used previously to evaluate PH. Qualitatively assessing flow patterns in the MPA, Reiter *et al.*[24] reported that in patients with pulmonary arterial hypertension abnormal separation between the boundary layer next to the wall of the MPA resulted in abnormal vortex development in the MPA. In addition, they reported delayed acceleration times in the MPA [24]. These hemodynamic phenomena should influence PA-RV interactions by changing both PVR and RV function. Interestingly, we did not detect a significant difference in time-to-peak acceleration. In fact, we found an earlier time to peak flow after pulmonary artery embolization and induction of PH. The different observations between this study and that of the Reiter group are presumably related to differences in the etiology of PH, acute in the current study and chronic in the study by Reiter *et al.*[24].

A benefit of using 4D flow CMR to quantify blood flow is that quantification can be performed of any vessel within the imaging volume *a posteriori*. In cases where flow quantification is performed in numerous vessels, the overall scan time is typically less using accelerated 4D flow CMR methods than using standard 2D flow CMR techniques. The stroke volumes measured with the 4D flow CMR flow data were slightly lower than the stroke volumes determined using Cine bSSFP. MPA flow volumes with 4D flow CMR were substantially lower than values observed with 2D flow CMR. Frydrychowicz *et al*. [15] also reported underestimation of Qp and Qs using the same 4D flow CMR study as that used in the current study. Some of these differences may be related to differences in temporal resolution and differences in flow during free breathing (4D flow) and end-expiration (2D flow).

Limitations of the current study include the fact that the reference standard used for LV volumes were axial CINE bSSFP images. Although the LV short-axis orientation is typically used to calculate LV volumes, Fratz *et al*. recently reported that axial images were more reproducible than short-axis images for calculating LV volumes in patients with repaired tetralogy of Fallot [25]. Future studies comparing short-axis reconstructed 4D flow CMR data to short-axis 2D CINE bSSFP will have to be conducted to confirm the small bias observed in this study.

Other limitations of this study include (a) a small sample size without a cohort to validate the model, (b) the estimation of the trans-pulmonary pressure gradient assumes that the modified Bernoulli equation is valid and requires the presence of tricuspid valve regurgitant jet, and (c) the fact that the equation for calculating PVR is based on RHC data obtained during acute embolic PH, which will most likely not be applicable to chronic PAH. In acute pulmonary embolism, right atrial pressures are elevated, reducing the RA-RV pressure gradient and, therefore, lowering the peak TRV. Although acute pulmonary embolism is the most common cause of acute PH [26], it is not clear from this study if these results would be transferable to acute pulmonary thromboembolism in humans or to other etiologies of PH. Subsequent studies will have to be conducted to assess the relationship between TRV/Q_P_ in other causes of PH, including in humans.

## Conclusions

In conclusion, this study demonstrates the feasibility of comprehensively estimating pulmonary vascular resistance, assessing ventricular function and pulmonary artery hemodynamics in a canine model of acute embolic PH using 4D flow CMR. Although not explored in this study, another potential benefit of using 4D flow CMR to assess PH is the derivation of additional hemodynamic parameters such as wall shear stress [16,18,27,28] and pulse wave velocities [29,30], which could provide further insights into pulmonary artery remodeling and interactions between pulmonary arterial stiffening and RV dysfunction.

## Competing interests

This study was supported by funding from the following funding sources: National Institutes of Health: R01HL072260, R01HL105598, R01HL086939.

Department of Radiology Research and Development Fund.

The project was supported by the Clinical and Translational Science Award (CTSA) program, previously through the National Center for Research Resources (NCRR) grant 1UL1RR025011, and now by the National Center for Advancing Translational Sciences (NCATS), grant 9U54TR000021.

A.F. received an educational stipend from Bracco Diagnostics.

The Departments of Radiology and Medical Physics receive support by GE Healthcare.

## Authors’ contributions

AR – Study design, data acquisition and analysis, manuscript preparation and editing. AF – Study design, data acquisition, manuscript editing. KMJ – Study design, manuscript editing. HK – Study design, data acquisition, manuscript editing. NCC – Study design, data acquisition, manuscript editing. OW – Study design, manuscript editing. CJF – Study design, data acquisition and analysis, manuscript preparation and editing. All authors read and approved the final manuscript.
